# Exploring Factors Influencing Nursing Task Prioritization for Supportive Information System Design: Qualitative Study With Thematic Analysis

**DOI:** 10.2196/89940

**Published:** 2026-06-05

**Authors:** Kodai Iwamoto, Mayumi Toyama, Goshiro Yamamoto, Chang Liu, Kazumasa Kishimoto, Yukiko Mori, Tomohiro Kuroda

**Affiliations:** 1 Department of Informatics, Graduate School of Informatics, Kyoto University, 54 Shogoin-kawahara-cho, Sakyo-ku, Kyoto, Japan, 81 75 366 7701; 2 Department of Health Informatics, Graduate School of Medicine, Kyoto University, Kyoto, Japan; 3 Preemptive Medicine and Lifestyle Related Disease Research Center, Kyoto University Hospital, Kyoto, Japan; 4 Department of Real World Data R&D, Graduate School of Medicine, Kyoto University, Kyoto, Japan; 5 Division of Medical Information Technology and Administration Planning, Kyoto University Hospital, Kyoto, Japan

**Keywords:** nursing task prioritization, computational tractability, semistructured interview, thematic analysis, novice nurses

## Abstract

**Background:**

Nurses are required to perform multiple tasks concurrently, which leads to multitasking situations, and they have to continuously determine which tasks should be prioritized. This is particularly challenging for novice nurses. Although IT-based systems supporting prioritization have begun to emerge, research on the types of information required when prioritization is processed computationally is scant. Despite the clear need for a supportive information system to assist nursing task prioritization, such systems are not yet sufficiently developed.

**Objective:**

This study aimed to explore the appropriate granularity and structure of information that should be provided to computational systems to support decision-making based on the influencing factors of nursing task prioritization.

**Methods:**

Semistructured interviews were conducted with 10 nurses working in general wards to examine the factors they consider when determining task prioritization during clinical practice. Data were analyzed using an inductive, semantic approach based on a thematic analysis framework.

**Results:**

Three themes and nine categories including (1) medical condition assessment factors (signs of acute physiological changes and indicators of clinical status and conditions), (2) patient-related nursing care factors (physical status, psychological condition, and personal characteristics; care needs during hospitalization; and treatment goals and care preferences), and (3) organizational and operational work factors (temporally structured tasks, requiring collaboration partners for task execution, environmental factors affecting task performance, and institutional- and ward-level policies) were identified.

**Conclusions:**

Analysis of computational tractability of the identified factors indicated that medical condition assessment factors are relatively quantifiable. In contrast, patient-centered care and organizational and operational work factors rely on contextual and experiential judgment, limiting standardization and formalization. Regarding such ambiguous and context-dependent elements, flexible information-processing approaches, such as large language models, in addition to conventional rule-based methods, may be effective. Furthermore, the appropriate level of information granularity should be determined by the nature of the prioritization outputs required in actual nursing practice rather than the degree of abstraction itself.

## Introduction

### Background

Clinical nurses care for multiple patients while concurrently performing a wide range of patient-specific nursing tasks. Consequently, multiple tasks frequently overlap within the same time frame, a phenomenon commonly defined as multitasking—the concurrent execution of 2 or more tasks [1,2]. Multitasking is considered among the most prevalent stressors in nurses’ daily practice [3] and has been reported to occur in approximately 26%‐50% of nursing activities [1,2,4]. Frequent multitasking is associated with task interruptions [5], which in turn increase cognitive and mental workload, leading to deviations from standard procedures, task errors, and increased risk of burnout [6-10].

Such challenges extend beyond interruptions, persisting throughout nursing care delivery. Particularly, when multiple tasks must be performed concurrently, nurses must determine the tasks that should be prioritized. In Japan, nursing students typically care for only one patient during clinical training, limiting their exposure to multitasking situations. Consequently, novice nurses often struggle to prioritize care after entering clinical practice [11-13]. Although prioritization challenges affect all nurses, they tend to be pronounced in early professional practice.

Nursing prioritization is the ordering of nursing problems by urgency and/or importance [12]. However, in clinical practice, prioritization is not determined by a single criterion; rather, it is influenced by multiple factors, including patient acuity and care intensity, availability of resources, organizational and personal values, nursing theories and models, and nurses’ proficiency [12,14]. Additionally, prioritization is influenced by multiple mechanisms, including the Maslow effect (prioritization of life-sustaining and physical care), the outcome effect (immediate visibility of intervention outcomes), the time-cost effect (short or more predictable tasks), the collective adaptation effect (tasks emphasized within the team), and the audit effect (tasks confirmed by physicians or managers) [15]. These findings indicate that nursing prioritization is complex, shaped by multiple, overlapping cognitive and organizational factors rather than a single criterion.

Previous studies have introduced educational interventions using simulations and virtual reality to enhance prioritization skills [16-18]; however, their effectiveness has not been consistently demonstrated. Although real-time role modeling by educators or senior nurses is effective [19], such approaches rely heavily on individual interpretation and experiential learning, limiting their applicability as clinical decision support. In contrast, IT-based approaches, such as rule-based methods that identify relevant factors using logistic regression and construct decision trees for prioritization, have also been proposed [20]. While useful in specific applications such as staff scheduling [21], these approaches face significant challenges owing to implicit, context-dependent conditions that are difficult to formalize mathematically [22]. Even in relatively stable task environments, adapting rule-based methods to real-world clinical practice remains challenging. Although previous studies have attempted to identify and organize detailed nursing elements into rule-based frameworks, the diversity and context-dependence of practice hinder the representation of all relevant factors as explicit conditions. Moreover, only a few studies have systematically examined how factors influencing nursing prioritization can be structured for computational processing, including the level of information granularity and structure of representation that enable such processing [12,14,15,20]. Thus, despite the clear need for a supportive information system to assist nursing task prioritization in clinical practice, such systems remain insufficiently developed. This gap can be addressed by acquiring a clearer understanding of how nurses’ prioritization decisions can be represented in a form suitable for computational handling.

### Aim

This study aims to explore how nursing prioritization factors can be represented in terms of information granularity and structure when computational processing is assumed. To this end, thematic analysis was used to identify and organize the influencing factors of nurses’ prioritization decisions. By elucidating the information structures suitable for computational handling, this study provides foundational insights for developing information systems that support nursing prioritization.

## Methods

### Study Overview

In this qualitative study, semi-structured interviews and a thematic analysis were conducted. This study was conducted in accordance with the Consolidated Criteria for Reporting Qualitative Research checklist.

### Setting and Sampling

Participants were recruited between September and November 2024 from general wards of a national university hospital with a nurse-to-patient ratio of at least 1:7. As factors influencing nursing prioritization may differ by ward type and years of nursing experience, both novice and experienced nurses working in general wards were included. Novice nurses included those with 2 or fewer years of clinical experience, whereas experienced nurses included those with 5 or more years of experience and at least 1 year of service in their current ward. Both wards used the partnership nursing system as their nursing care delivery model. Potential participants were identified through the researchers’ professional networks in collaboration with ward nurse managers, and purposive sampling was used. All candidates received an explanation of the study purpose and procedures, including audio recording; those who agreed to participate provided written informed consent.

### Data Collection

The interviews were conducted once, face-to-face in Japanese by a single researcher (KI, registered nurse; male) with experience in qualitative research, and took place in the participants’ wards. Individual, semistructured interviews were conducted, with primary questions focusing on factors influencing task prioritization in nursing practice; these questions were generated through a pilot study. Follow-up questions were asked as appropriate based on the participants’ responses. The order and content of the questions were flexibly adjusted. Multimedia Appendix 1 provides an interview guide. Data collection was concluded when no new categories were generated. The interviewer and participants had previous professional contact through prior research activities. Field notes were taken during the interviews to supplement contextual understanding.

### Data Analysis

This study aims to identify the factors influencing nursing task prioritization and elucidate context-dependent, multifaceted patterns of meaning. Therefore, an inductive and semantic approach to thematic analysis, as proposed by Braun and Clarke [23], was used to extract meaning from the data.

The analysis involved the following steps. First, the audio-recorded interviews were transcribed verbatim, and the transcripts were read repeatedly to achieve familiarization with the data. Subsequently, the researcher (KI) independently generated the initial codes inductively by focusing on meaningful units within the data. Similarities among the generated codes were examined, and themes and categories were identified. The identified themes and categories were reviewed collaboratively with another researcher (MT; female), and iterative refinement, including merging and differentiating themes, was conducted until consensus was reached. Finally, each theme and category was clearly defined and named to capture its essential meaning.

To enhance the credibility of the analysis, interpretations were discussed among researchers from different academic backgrounds. To reduce bias from the researcher’s professional nursing background, ongoing reflexive discussions were held throughout the analytical process, and guidance was sought from an expert in thematic analysis. To further ensure credibility, transcripts were returned to the participants, and some participants confirmed that the content was accurate. Considering participant burden, feedback on the final analytical results was not requested. All analyses were conducted in Japanese, and the findings were translated into English. The researchers reviewed the translations to ensure accuracy. The data were analyzed using Microsoft Excel (version 2511).

### Ethical Considerations

This study was approved by the Kyoto University Graduate School and Faculty of Medicine Ethics Committee (approval 4460). Participation in the study was voluntary. Recorded audio data were processed on devices with appropriate security measures, and participant privacy was protected through pseudonymization during the generation of analysis datasets. All participants were provided with a detailed explanation of the study, and written informed consent was obtained prior to participation. Participants were also informed of their right to withdraw consent at any time. No compensation was provided for participation in the study.

## Results

### Participant Characteristics

Table 1 presents details of the study participants. The participating wards included cardiology and thoracic surgery; 4 experienced and 6 novice nurses were recruited from the cardiology and thoracic surgery wards, respectively, resulting in 10 interviews. The mean interview length was 52.0 (SD 9.6) minutes. No participants declined to participate or withdrew from the study.

**Table 1.** Demographic profile of the participants.

Participants	Groups	Sex	Years of experience		Ward type
			Nursing	Ward	
1	Veteran	Male	9th year	5th year	Cardiology
2	Veteran	Male	15th year	3rd year	Cardiology
3	Veteran	Female	6th year	6th year	Cardiology
4	Veteran	Female	13th year	3rd year	Cardiology
5	Novice	Female	1st year	1st year	Thoracic surgery
6	Novice	Female	2nd year	1st year	Thoracic surgery
7	Novice	Female	2nd year	2nd year	Thoracic surgery
8	Novice	Female	2nd year	2nd year	Thoracic surgery
9	Novice	Female	2nd year	2nd year	Thoracic surgery
10	Novice	Female	2nd year	2nd year	Thoracic surgery

### Overall Theme

#### Overview

Table 2 summarizes the factors influencing task prioritization by the general ward nurses. Three overarching themes including (1) medical condition assessment factors, (2) patient-related nursing care factors, and (3) organizational and operational work factors were identified. These themes comprised 9 categories.

**Table 2.** Themes, categories, and codes identified through thematic analysis.

Themes and categories	Codes
Theme A: medical condition assessment factors	
A-1: signs of acute physiological changes	Deterioration of hemodynamic status (eg, onset of arrhythmia and sudden decrease in blood pressure), worsening of respiratory status (eg, sudden decrease in SpO_2_)
A-2: indicators of clinical status and conditions	Medical interventions (surgery, medication therapy, medical procedures, and diagnostic tests), laboratory data, vital signs, clinical findings, clinical symptoms, indwelling devices, oxygen therapy, medication therapy, fluid balance, progress notes, changes in condition during hospitalization
Theme B: patient-related nursing care factors	
B-1: physical status, psychological condition, and personal characteristics	Activities of daily living, presence of fall risk, physiological function (ability to expectorate), presence of dementia, presence of agitation, communication ability, personality traits
B-2: care needs during hospitalization	Illness stage, infection presence, intrahospital transfer, assistance with personal grooming, assistance with medication management, response to nurse call systems
B-3: treatment goals and care preferences	Discharge preparation, do not resuscitate orders, understanding of end-of-life preferences
Theme C: organizational and operational work factors	
C-1: temporally structured tasks	Time-specific tasks, non–time-specific tasks, unscheduled tasks, sequence-dependent tasks
C-2: required collaboration partners for task execution	Doctor, nurse, allied health professionals, family
C-3: environmental factors affecting task performance	Physicians' availability, nursing staff resources, temporal working conditions, task complexity, clinical procedures volume, number of assigned patients, location of assigned patients, presence of primary patients
C-4: institutional- and ward-level policies	Within the hospital, within the ward

#### Theme A: Medical Condition Assessment Factors

##### Overview

This theme represents factors that nurses consider when evaluating patient acuity and prioritizing tasks based on signs of acute physiological changes and clinical indicators. Nurses assessed and integrated information ranging from life-threatening changes to indicators requiring continuous monitoring to guide prioritization decisions. This theme comprises the following 2 categories.

##### A-1: Signs of Acute Physiological Changes

This category reflects situations in which nurses detect acute and marked changes in physiological indicators, such as the onset of arrhythmias, sudden decreases in blood pressure, or abrupt drops in oxygen saturation, and recognize these changes as signals of clinical deterioration requiring immediate attention. Such indicators strongly influence nurses’ decisions to prioritize specific nursing tasks.

If an arrhythmia appears on the ECG or the oxygen saturation drops, I think I would prioritize going to see the patient.

[Participant 1, veteran]

If there’s a significant drop in blood pressure, I feel that it takes priority over scheduled tasks.

[Participant 6, novice]

##### A-2: Indicators of Clinical Status and Conditions

This category encompasses information reflecting the patient’s current condition, including laboratory data, vital signs, and clinical findings and symptoms, as well as factors related to ongoing medical management, such as medical interventions, indwelling devices, medication therapy, and oxygen therapy. The findings showed that nurses comprehensively integrate these diverse indicators to assess patient stability and care needs and to adjust task prioritization accordingly.

Patients who have just undergone surgery, those on the first postoperative day, those on mechanical ventilation, those receiving oxygen therapy, or those with unstable vital signs—I tend to prioritize seeing those patients first.

[Participant 8, novice]

Patients with higher severity often have multiple infusions, drains, or chest tubes in place, and sometimes temporary pacing leads as well. I assess those factors because their circulatory status can become quite unstable.

[Participant 2, veteran]

### Theme B: Patient-Related Nursing Care Factors

#### Overview

This theme reflects how nurses prioritize nursing tasks based on patient-specific factors including physical status, psychological condition, and personal characteristics; care needs during hospitalization; and treatment goals and care preferences. Nurses consider not only patients’ medical conditions but also their functional status, psychological state, and understanding of and attitudes toward treatment when prioritizing tasks according to the clinical situation. This theme comprises the following 3 categories.

#### B-1: Physical Status, Psychological Condition, and Personal Characteristics

This category reflects evaluations based on individual patient characteristics, including physical and physiological functions, such as activities of daily living and the ability to expectorate, as well as psychological characteristics, such as cognitive function, presence of agitation, communication ability, and personality traits. The findings showed that nurses adjusted task prioritization and care approach according to the necessity of care, intervention complexity, and monitoring requirements based on these characteristics.

About patients with dementia, there is often a greater need for assistance with ADLs, and in terms of building trust or communication, I tend to go to them first. If I am late, even patients with dementia can feel anxious, stressed, or irritated, so I try to attend to them early.

[Participant 4, veteran]

Because they cannot express themselves verbally, I think they should be prioritized in case something urgent happens.

[Participant 6, novice]

#### B-2: Care Needs During Hospitalization

This category indicates that prioritization was determined based on care needs arising from patients’ medical conditions and daily living during hospitalization. Nurses considered clinical assessments such as illness stage and presence of infection, alongside patients’ expressed needs related to daily living support, including hospital transfer, personal grooming, and medication management. Furthermore, responses to nurse call systems were prioritized, as they were recognized as directly influencing patients’ sense of security and the establishment of trust (Textbox 1).

Textbox 1. **Representative quotes related to Category B-2**

“In the hyperacute phase, that is the highest priority. For acute and terminal phases, it may depend on the individual’s condition. Even among terminal patients, there are those whose condition is relatively stable and those who are much closer to death, and in those cases, they may be prioritized at the same level as hyperacute patients. Patients in a stable phase would come later” [Participant 4, veteran].

“When patients ask to go shopping or to be taken to the post office, I try not to postpone it too much. It’s also about the relationship—if you make them wait too long, they become anxious, so I try to be mindful not to keep them waiting for too long” [Participant 3, veteran].

“For nurse call responses, I basically go as soon as it rings, unless there is a very compelling reason not to. In principle, I will respond immediately” [Participant 9, novice].

#### B-3: Treatment Goals and Care Preferences

This category indicates that factors related to patients’ values and decision-making, such as their treatment goals and care preferences, influenced prioritization. Information related to treatment direction, including discharge preparation, do-not-resuscitate orders, and understanding of end-of-life preferences, was shown to be a key factor influencing nurses’ decisions regarding care timing and approach (Textbox 2).

Textbox 2. **Representative quotes related to Category B-3**

“I think we start gathering information early on about how the patient wants to live after discharge, but we also check how they feel at that moment, given their current condition” [Participant 1, veteran].

“For terminal patients, it depends on the severity. If it’s really down to just a few days or even that day, then the priority becomes very high. But it also changes depending on whether a DNR has been established or not” [Participant 6, novice].

“For patients receiving best supportive care, if they express how they want to proceed from here, I try to listen to that, and if I can make time during the day, I go to see them” [Participant 7, novice].

### Theme C: Organizational and Operational Work Factors

#### Overview

This theme focuses on organizational and operational conditions under which tasks are performed, highlighting structural and environmental factors influencing planning, execution, and prioritization. Beyond clinical assessments and patient-related factors, nurses adjusted priorities realistically and flexibly by considering temporally structured tasks, required collaboration partners for task execution, environmental factors affecting task performance, and institutional- and ward-level policies. This theme comprises the following 4 categories.

#### C-1: Temporally Structured Tasks

This category reflects task characteristics with temporal constraints, including tasks with predefined timing or sequence of execution, and those requiring situational adjustment. Nurses differentiated tasks that must be performed at specific times, such as medication administration, flexibly timed tasks, and unscheduled tasks. They prioritized tasks based on overall workflow, while also planning tasks that required a specific execution sequence according to their impact on subsequent activities (Textbox 3).

Textbox 3. **Representative quotes related to Category C-1**

“I make sure to go at 9 o’clock for patients who have scheduled oral medications, since the administration time is fixed” [Participant 5, novice].

“For tasks that are already scheduled, such as hygiene care or wound care, I adjust my schedule so that they can be completed in the morning” [Participant 3, veteran].

“I often have to hurry to make sure blood glucose measurements are done in time before meals” [Participant 10, novice].

#### C-2: Required Collaboration Partners for Task Execution

This category reflects the individuals with whom nurses coordinate care as part of their duties, including doctors, other nurses, allied health professionals, and families involved in clinical decision-making or task execution.

For procedures, when a physician has a fixed schedule—such as outpatient clinics or surgeries—and there is no flexibility around that timing, we adjust other tasks so that we can carry them out accordingly.

[Participant 4, veteran]

For admissions, we often do them together as a pair, because there are records to complete and one person may need to explain things while the other documents, so admissions are often handled by two nurses.

[Participant 4, veteran or novice]

#### C-3: Environmental Factors Affecting Task Performance

This category indicates that the human, physical, and temporal environments surrounding nurses influence task execution and prioritization. Nurses adjusted their work based on human resource constraints such as physician availability, nursing staff resources, and shift schedules, and environmental factors, including clinical procedure volume, task complexity, and patient numbers and locations. These factors resulted in variations in task prioritization depending on the day of the week or time of day. In situations where staffing capacity allowed greater flexibility, tasks that were usually deprioritized were sometimes addressed.

On weekends, since there are fewer examinations, the ward tends to be calmer, so we try to focus on things like giving bed baths to patients who require full assistance or shampooing patients who haven’t been able to get it done.

[Participant 6, novice]

When I have fewer assigned patients and a new admission comes in, I try to use that time to provide orientation for the patient, such as explaining the ward routines.

[Participant 10, novice]

#### C-4: Institutional- and Ward-Level Policies

This category indicates that institutional- and unit-level policies and operational rules influence how nursing tasks are performed, including the timing of care delivery and decisions regarding task prioritization.

Visiting hours start at 2:00 p.m., and since that is a hospital-wide rule, we provide guidance or education to family members after they arrive.

[Participant 1, veteran]

For medications such as immunosuppressants, blood concentration levels are important, so we make sure they are administered at the designated time. We try to administer them as early as possible and confirm adherence—this is something like a ward-level rule.

[Participant 8, novice]

## Discussion

### Principal Findings

#### Overview

This study identifies 3 themes and 9 categories influencing nurses’ task prioritization in general wards. Theme A represents medical urgency and acuity, theme B reflects patient individuality and values, and theme C encompasses organizational and operational constraints. Consistent with previous studies, the findings suggest that nurses’ prioritization is a dynamic process integrating multiple elements according to the situational context [12,14,15]. Regarding differences in information structure across themes, the elements in theme A are relatively amenable to simple classification (eg, binary or categorical) or quantification, whereas aspects of themes B and C rely on situational interpretation and experiential judgment, complicating uniform representation.

#### Theme A: Medical Condition Assessment Factors

The elements in theme A were largely consistent with previously reported criteria for medical urgency and acuity. Regarding A-1, previous studies have shown signs of acute physiological changes such as dyspnea, chest pain, airway obstruction, stroke, and massive hemorrhage to require immediate, high-priority intervention [14,24-26]. Similarly, the indicators of clinical status and conditions in A-2 were aligned with those reported in earlier research, including medical interventions, laboratory data, vital signs, oxygen therapy, medication therapy, indwelling devices, clinical symptoms, and progress notes [14,15,27]. In contrast, elements such as clinical findings and fluid balance were uniquely identified. These factors may previously have been subsumed under broader concepts such as “physiological needs” or “patient assessment,” and therefore not explicitly defined as discrete elements [15,27]. Particularly, fluid balance appears to be a context-dependent factor influenced by the specific clinical setting of a cardiology ward. This finding highlights a key challenge: as elements are identified in greater detail, context-specific judgment criteria increase, potentially complicating comprehensive organization and standardization.

#### Theme B: Patient-Related Nursing Care Factors

In theme B, judgment elements based on patient individuality and values were identified. Regarding B-1, physical status, psychological condition, and personal characteristics such as activities of daily living, dementia, agitation, and personality traits have also been reported in previous studies [14,25,28,29]. While some studies emphasize that psychological support should be prioritized [14], others highlight that such care is often left unfinished because of time constraints and difficulties in evaluating outcomes [15]. Although these factors can guide prioritization, their weighting likely depends heavily on individual nurses’ values and clinical experience [12]. Furthermore, previous research shows that early-career nurses tend to emphasize psychosocial needs, whereas experienced nurses tend to deprioritize them or consider them the responsibility of other professions [28]. Physiological functions such as the ability to expectorate (ie, clear sputum from the airway) and communication were newly identified in this study and are likely influenced by the thoracic surgery ward study setting. Regarding B-2, care needs during hospitalization, such as the presence of infection and responses to nurse call requests, have been reported in previous studies [15,24]. In contrast, factors including illness stage, intrahospital transfers, assistance with personal grooming, and medication management were newly identified in this study. These elements reflect patient-specific needs and, compared with the medical factors in theme A, may be less likely to become explicit in the prioritization decision-making process. Regarding B-3, treatment goals and care preferences, previous studies show that discharge support and planning are often incomplete [15]. In contrast, this study identifies discharge preparation as a prioritized element. This difference is attributable to the target hospital, where the partnership nursing system has been implemented, suggesting that a care delivery model with relatively sufficient human resources may influence nurses’ prioritization decisions. These findings primarily reflect what should be prioritized based on patient-specific considerations.

#### Theme C: Organizational and Operational Work Factors

In theme C, judgment elements based on organizational and operational constraints were identified. In contrast to theme B, these factors primarily constrain what can be feasibly performed in practice. Regarding C-1, temporally structured tasks were consistent with previous studies, showing that, during physical emergencies, routine tasks are interrupted to provide immediate care [28], corresponding to “unscheduled tasks” in this study. In contrast, few previous studies have clearly distinguished between time-specified, non–time-specified, and sequence-dependent tasks based on time constraints or execution order. Although this represents a highly detailed classification, it reflects the level of granularity required for computational processing. The elements related to C-2, which required collaborators for task execution (doctors, nurses, allied health professionals, and family members), were newly identified in this study. Although these elements may not directly determine task priority, they represent key influencing conditions for task feasibility. Regarding C-3, environmental factors affecting task performance, such as human resources, task difficulty, procedure volume, and number of assigned patients, have also been reported in previous studies [12,14,27-30]. In contrast, factors including physician availability, day of the week, location of assigned patients, and whether the patient was assigned as a primary patient were specific to this study. Differences in the implementation of examinations and rehabilitation depending on the day of the week may influence the structure of prioritization, suggesting that prioritization depends on institutional characteristics. Regarding C-4, institutional- and ward-level policies, previous studies show that ward philosophy and the nursing theories or models adopted influence prioritization decisions [12,15,24,27]. Given that nursing practices are performed within specific environments, context-dependent judgment criteria were identified. The elements identified in C-2, C-3, and C-4 are suggested to serve both mediating and moderating roles in task prioritization. Specifically, they mediate the formation of prioritization judgments while also moderating the strength and direction of these effects. For example, nursing practice is not uniform; the priority assigned to the same task varies across settings, such as hospitals and wards, long-term care facilities, and home care. Furthermore, the range of feasible tasks is constrained by factors such as interprofessional collaboration and staffing conditions, which in turn alter the scope of tasks that can be prioritized. Taken together, these findings suggest that the elements identified in themes B and C can be understood as different dimensions of contextual judgment, encompassing both patient-specific and organizational factors that shape prioritization decisions.

Building on these functional roles, the following discussion further conceptualizes organizational context as a broader mechanism underlying task prioritization. Although this study does not address the attributes of the requester, previous research shows that in nonhospital settings, requests from managerial staff are more likely to be prioritized [12,28]. In contrast, in general hospital wards, nurses primarily assume leadership in task execution. This contrast suggests that the influence of requester attributes is contingent on organizational context, particularly differences in roles, authority structures, and chains of command. These findings suggest that ward characteristics function as a foundational mechanism structuring task prioritization processes. Specifically, such characteristics encompass not only explicit operational rules, such as visiting hours, but also the configuration of stakeholders and their power dynamics. Through their interaction, these factors shape nurses’ subjective judgment in practice. Furthermore, as previous studies have shown, nurses tend to prioritize physicians’ expectations due to the nature of their roles, indicating that task prioritization is conducted with close attention to established chains of command [26]. Additionally, previous studies indicate that nurses’ subjective assessments—such as the time required to complete a task and the predictability of intervention outcomes—as well as value-based considerations, including the prioritization of physician expectations and tasks that emphasize human dignity, also influence prioritization decisions [15,26]. Taken together, these findings suggest that task prioritization is shaped by a complex interplay of organizational, contextual, and individual factors that are difficult to fully formalize, including nursing theories and judgments based on individual values and professional experience.

### Characteristics of the Extracted Factors and Their Computational Tractability

This study explores which information structures are amenable to computational processing, based on the elements identified through thematic analysis, and conceptualizes mechanisms for supporting nursing task prioritization.

Regarding information processing approaches for prioritization, both rule-based methods, such as integer linear programming, and non–rule-based approaches, such as large language models (LLMs), can be considered. ILP enables the derivation of optimal solutions through the explicit formulation of objective functions and constraints; however, it is limited in adequately representing the ambiguous and context-dependent elements inherent in real-world clinical practice. Additionally, its high computational cost and the need for recalculation in dynamically changing environments pose practical challenges for real-world implementation. In contrast, LLMs can process information that includes implicit judgments and ambiguous expressions that are not explicitly defined by rules. This characteristic suggests their potential usefulness in handling the tacit knowledge and context-sensitive decision-making in nursing practice. Nevertheless, LLMs present challenges, including the opacity of their reasoning processes and concerns regarding explainability, safety, and reliability when applied to medical information.

In routine clinical practice, nurses’ decision-making is typically grounded in higher-level, abstract categorical understanding, while drawing on more concrete, code-level information. In contrast, from the perspective of computational information processing, even code-level data can be handled in a formalized manner. However, the codes identified in this study do not fully encompass all factors influencing nurses’ decisions. Particularly for highly context-dependent elements, explicit structuring remains a challenge. Specifically, the elements within theme A (medical condition assessment factors) are relatively capable of simple classification (eg, binary or categorical) or quantification, whereas aspects of theme B (patient-related nursing care factors) and theme C (organizational and operational work factors) require situational interpretation and experiential judgment. Consequently, these elements cannot be easily represented within a uniform information structure. This structural heterogeneity highlights the difficulty of defining a single, standardized level of granularity or representation for information used in prioritization. Together, the key challenges identified in this study can be summarized as how to address the long-tail structure of decision-making factors and how to determine the appropriate level of granularity for information used in prioritization. As a complementary approach to these challenges, integrating category-level information through retrieval-augmented generation using LLMs may enable the partial incorporation of contextual variability and judgmental nuances that are difficult to capture using conventional rule-based or mathematical optimization methods. Consequently, while defining information at a highly detailed level may limit the comprehensiveness of captured judgment factors, excessive abstraction may render the information too vague for LLMs to interpret in a manner consistent with the intended decision-making process. Therefore, the granularity of information should be determined by the nature of the prioritization outputs required in clinical practice rather than its level of detail.

The focus of this study—prioritization of nursing tasks—can be situated within the framework proposed by Barrett and Jones [31], which conceptualizes 3 types of nurse-artificial intelligence interaction: task augmentation, task automation, and hybrid. Specifically, this study is positioned as an attempt toward task automation [31]. We posit that achieving full automation requires that all elements involved in prioritization decisions be represented in a structured and computable form. However, our findings indicate that the factors underlying task prioritization can be broadly categorized into those that are amenable to quantification and structuring and those that rely on unstructured information such as natural language. This distinction is consistent with the framework proposed by Autor et al [32], in which routine tasks are more susceptible to substitution by computer technologies, whereas nonroutine tasks tend to be complemented rather than replaced. In this context, elements that can be structured may be processed using rule-based approaches, whereas elements that resist structuring may require interpretation through generative artificial intelligence or may be difficult to incorporate into computational processes altogether. These unstructured elements are characterized by their reliance on context-dependent judgment, and their structuring becomes even more challenging when incorporating not only clinical information but also patient preferences and needs. The difficulty of structuring such elements reflects the inherent ambiguity of task characteristics in the nursing domain, suggesting that it is not feasible to fully structure all relevant information. Therefore, the findings of this study highlight the importance of designing and implementing technologies under the assumption that complete structuring is unattainable. Similar to the indispensable role of free-text fields in electronic health records, our results underscore the need for system designs that accommodate both structured and unstructured information.

### Limitations

This study has several limitations. First, the codes identified as influencing prioritization may not comprehensively capture all the elements that affect nurses’ decision-making. Second, the relative influence of each factor was not quantitatively evaluated; therefore, the relative importance and potential interactions among factors remain unclear. Third, the findings were derived from a specific context and population, and the results may not be generalizable to settings with different ward characteristics, organizational structures, or nursing systems.

### Future Directions

#### Overview

The system envisioned in this study is designed to support the prioritization of nursing tasks and to partially automate the associated information-gathering processes. Such a system may be implemented via a smartphone-based interface that presents the next task to be performed and allows nurses to record task completion through simple interactions. It may be used both before the start of a shift to provide an overview of the day’s workload and continuously during the shift to accommodate changes in clinical conditions. The future research required for the development and implementation of this system is outlined below.

#### Technical Validation of Prioritization Criteria

To translate the findings into rule-based information processing approaches such as ILP, further examination regarding which codes should be incorporated into prioritization systems, the appropriate level of granularity, and how the weighting of each code should be determined is required. Additionally, it is necessary to investigate which levels of abstraction (eg, code or category) and what types of information are most effective when presenting prioritized recommendations to nurses in clinical practice.

#### Design of Supportive Information System

Previous research shows that nurses tend to undertake nonnursing tasks and that such tendencies are associated with an increased likelihood of nursing tasks being left undone (NTLU) [33]. Moreover, experiences of NTLU have been reported to be strongly associated with job dissatisfaction than performing nonnursing tasks [33]. Importantly, NTLU often involves intrinsically meaningful activities, such as comfort or talk with patients, which are prone to deprioritization under time pressure [28,33]. This phenomenon may be partly explained by the mere-urgency effect, whereby urgent tasks are prioritized over those that are more important but less time-critical, thereby hindering appropriate prioritization [34].

Accordingly, the design of support information systems should not simply aim to mechanistically determine or present task priorities. As suggested by prior research proposing a 7-stage normative framework for prioritizing nursing activities, they should support nurses in making informed decisions while preserving professional values and the core aspects of nursing practice [35]. In other words, instead of immediately reprioritizing nonurgent interruptions, decision support systems should facilitate judgments that consider job satisfaction, nursing care quality, and sustainability of “nursing as a profession.”

### Conclusions

Although there is a clear need for a supportive information system for nursing task prioritization, such systems remain insufficiently developed. Accordingly, this study explored how factors influencing nursing prioritization can be represented in terms of information granularity and structure.

Thematic analysis was used to identify and organize the factors guiding nurses’ prioritization decisions. The results indicate that nurses’ prioritization constitutes a multilayered decision-making process comprising three qualitatively distinct themes: (1) objectively observable elements such as medical urgency and acuity, (2) context-dependent factors grounded in patient individuality and values, and (3) environmental factors including time constraints, staffing resources, and organizational policies.

Theme A (medical condition assessment factors) was relatively amenable to quantification. In contrast, themes B (patient-related nursing care factors) and C (organizational and operational work factors) required context-sensitive and experiential judgment. While these elements enable flexible, patient- and institution-specific decision-making, they complicate standardization and formalization. Accordingly, for such ambiguous and context-dependent elements, flexible information-processing approaches, such as LLMs, may be effective in complementing conventional rule-based methods. Furthermore, the appropriate level of information granularity should be determined by the nature of the prioritization outputs required in actual nursing practice, rather than by the degree of abstraction alone.

## Supplementary material

10.2196/89940 Multimedia Appendix 1 Interview guide.

## Abbreviations

ILP: integer linear programming
LLM: large language model
NTLU: nursing task being left undone
