# Supplementary material for: Exploring Factors Influencing Nursing Task Prioritization for Supportive Information System Design: Qualitative Study With Thematic Analysis
Source: JMIR Hum Factors. 2026 Jun 5;13:e89940. doi: 10.2196/89940 (PMC13240637; doi:10.2196/89940)
Supplement: Multimedia Appendix 1 [file humanfactors-v13-e89940-s001.docx]

1. What types of nursing tasks do you tend to prioritize when providing care?

1. Please list as many reasons as possible for prioritizing these tasks.
2. Which of these reasons do you personally consider most important, and why?
3. Do the tasks you prioritize change depending on the patient’s condition, diagnosis, or illness stage?
4. Do the tasks you prioritize vary according to the day of the week or time of day (e.g., shift)?
5. Do the tasks you prioritize change depending on the location of your assigned patients within the ward?
6. Does ward culture influence which tasks you prioritize?
7. Do changes in staffing levels (e.g., increase or decrease in the number of staff on duty) affect which tasks you prioritize?

2. What types of nursing tasks do you tend not to prioritize when providing care?

1. Please list as many reasons as possible for not prioritizing these tasks.
2. Which of these reasons do you personally consider most important, and why?
3. Do the tasks you do not prioritize change depending on the patient’s condition, diagnosis, or illness stage?
4. Do the tasks you do not prioritize vary according to the day of the week or time of day (e.g., shift)?
5. Do the tasks you do not prioritize change depending on the location of your assigned patients in the ward?
6. Does ward culture influence which tasks you do not prioritize?
7. Do changes in staffing levels (e.g., increase or decrease in the number of staff on duty) affect which tasks you do not prioritize?
